# PREDICTORS OF CHANGE IN CAROTID ARTERY ULTRASOUND MEASURES IN THE LONG LIFE FAMILY STUDY

**DOI:** 10.1093/geroni/igae098.3298

**Published:** 2024-12-31

**Authors:** Allison Kuipers, Caitlyn Kline, Ryan Cvejkus, Emma Barinas-Mitchell, Joseph Zmuda

**Affiliations:** University of Pittsburgh, Pittsburgh, Pennsylvania, United States; University of Pittsburgh, Pittsburgh, Pennsylvania, United States; University of Pittsburgh, Pittsburgh, Pennsylvania, United States; University of Pittsburgh, Pittsburgh, Pennsylvania, United States; University of Pittsburgh, Pittsburgh, Pennsylvania, United States

## Abstract

Early vascular changes of cardiovascular disease can be assessed using carotid ultrasound. The Long Life Family Study (LLFS) may help elucidate factors associated with decelerated vascular aging. Therefore, we identified predictors of change in carotid intima-media thickness (IMT) and interadventitial diameter (IAD) over ~6.0 years of follow-up (range 3.2-8.4) in 674 participants from the LLFS. Common carotid artery B-mode ultrasound was conducted using a GE LOGIQ 3 ultrasound system. We calculated change and annualized percent change (APCT) in mean carotid IMT and IAD. Age, sex, and study site were included in all models, and baseline measures of lifestyle, cardiometabolic conditions, and medication were tested in linear regression models (α=0.05). Final models were adjusted for relatedness using GEE. Participants were 67.5 years old (range 46-90), 43% male, 35% hypertensive, 6% diabetic, and 30% were on lipid-lowering medication, 96% didn’t smoke and 80% regularly walked >3hours/week. IMT increased 0.046mm or 1.08% annually, while IAD increased 0.166mm or 0.37% annually (all P< 0.0001). IMT APCT change was greater in those aged < 65 (1.37%) than over 65 (0.95%, P=0.004) but did not differ by sex (P=0.20). Whereas IAD APCT change did not differ by age group (P=0.10) but was greater in females (0.44%) than males (0.29%, P=0.006). Outside of age, sex, and site, independent predictors of IMT and IAD APCT included fasting glucose and smoking, plus triglycerides for IMT and systolic blood pressure for IAD. These findings highlight the need for continued research into the role of glucose homeostasis in vasculature aging.

